# Prevalence and Causes of Visual Impairment and Blindness in the Qassim Region, Saudi Arabia: A Retrospective Study

**DOI:** 10.7759/cureus.112786

**Published:** 2026-07-16

**Authors:** Abdulaziz H Alluhayb, Saud K Alamri, Ali S Alrashed, Nayef Alswaina

**Affiliations:** 1 College of Medicine, Qassim University, Buraydah, SAU; 2 Department of Ophthalmology, Qassim University, Buraydah, SAU

**Keywords:** blindness, ksa, prevalence, qassim region, visual impairment

## Abstract

Introduction: Visual impairment (VI) and blindness occur when processes and structures responsible for vision are malfunctioning or damaged. Neglect, inappropriate handling, and delayed treatment of this condition could lead to adverse visual health outcomes. Understanding the prevalence and causes of this condition will enhance preventative measures and ultimately reduce the number of occurrences.

Methods: The study was a retrospective cohort study that interrogated 385 medical records of patients of various age groups who attended outpatient eye clinics at Qassim University Medical City (QUMC) between January 2023 and December 2024. Data was first cleaned and then analyzed using IBM SPSS Statistics for Windows, Version 27 (Released 2019; IBM Corp., Armonk, New York, United States) to obtain crucial insights.

Results: The study found approximately 58.2% prevalence of VI and blindness among the patients. Most patients had moderate VI (123, 54.9%), followed by mild (70, 31.3%), severe (27, 12.1%), and blindness (4, 1.8%). The leading causes were refractive error (125, 32.5%) and cataract (80, 20.8%). Statistically significant associations were found between patients' nationality, presenting visual acuity (VA), systemic comorbidities, intervention, and the prevalence of VI and blindness (p=0.030, p<0.001, p<0.001, p=0.005). Intervention received by patients was positively associated with VI and blindness (AOR=3.40; 95% CI=1.219-9.498; p=0.019). Additionally, age (AOR=1.05; 95% CI=1.035-1.065; p<0.001) and presenting VA (AOR=0.42; 95% CI=0.231-0.756; p=0.004) were significantly associated with VI and blindness.

Conclusion: VI cases are prevalent among patients attending eye clinics at QUMC, presenting symptoms like decreased vision and blurred vision. The leading causes were refractive error and cataract, with most patients presenting moderate and mild VI. The most common comorbidities were diabetes mellitus and hypertension. There is a need for patients to take prompt action and preventive measures, including regular eye examinations, to minimize the incidence of VI and the risk of blindness.

## Introduction

Visual acuity (VA) refers to the sharpness of vision, indicating how clearly a person can distinguish fine details and object outlines. It represents the minimum distance at which two separate lines can still be identified as distinct by the viewer. In clinical settings, distance VA is typically assessed using Snellen charts, which are read from a standard distance of 6 meters (20 feet) [1]. Distance VA was assessed using the Snellen chart at a standard distance of 6 meters (20 feet), in accordance with the standard clinical protocol at our institution; illumination levels were not individually documented in the medical records reviewed (typically 160-200 lux), with patients tested monocularly at the designated distance and the smallest line read with fewer than two errors recorded as the VA level [1].

Visual impairment (VI) and blindness remain significant global public health challenges, arising from damage or dysfunction of ocular structures responsible for vision. These conditions negatively impact quality of life, reduce productivity, and increase healthcare burdens worldwide [2]. According to the World Health Organization (WHO), at least 2.2 billion people globally have a near or distance vision impairment, of whom at least one billion cases could have been prevented or have yet to be addressed [2]. Delayed diagnosis, neglect, or inadequate management often contribute to disease progression and irreversible visual loss [3]. Uncorrected refractive errors, cataracts, glaucoma, diabetic retinopathy, and corneal opacities are the leading contributors to VI globally [4].

However, there are regional differences due to variations in healthcare access, demographic characteristics, and socioeconomic factors. In Saudi Arabia, the burden of VI remains significant, with cataract and refractive errors consistently reported as the major causes in both national and regional assessments. A national analysis demonstrated that cataract continues to be the leading cause of blindness and VI across several regions of the country [5]. Likewise, a Riyadh-based clinical study identified a high prevalence of VI among adults, frequently associated with diabetes and other systemic comorbidities [6]. Unlike the study by Aldakhil et al. [7], which examined a rural community-based population in Qbah using community-level screening, the present study evaluated patients attending outpatient ophthalmology clinics at a tertiary referral hospital (Qassim University Medical City (QUMC)), representing a clinic-based population that may differ in disease severity, age distribution, and healthcare-seeking behavior [7]. The increasing burden of non-communicable diseases such as diabetes mellitus and hypertension in Saudi Arabia further compounds the risk of ocular complications, making the investigation of visual health trends within specific regions particularly important [6].

Therefore, this retrospective record-based cross-sectional study aimed to determine the clinic-based prevalence, documented causes, and factors associated with VI and blindness among patients attending ophthalmology outpatient clinics at QUMC between January 2023 and December 2024. The findings are intended to inform preventive strategies and improve clinical decision-making for eye care in the region.

## Materials and methods

Study design and setting

This retrospective record-based cross-sectional study was conducted at QUMC, a tertiary care institution located in Buraydah, Saudi Arabia. QUMC serves as a major referral center for ophthalmologic care in the region. All patients with complete medical records meeting the eligibility criteria who attended the ophthalmology outpatient clinics at QUMC during the 24-month study period were included, yielding a total of 385 eligible records.

Study population and sampling

A convenience sampling technique was used because only patients presenting to QUMC's outpatient ophthalmology clinics were accessible for record review, and the dataset was derived from routinely collected medical records, making systematic random selection across the broader Qassim population infeasible. This approach is consistent with similar retrospective clinical studies, such as that of Aldakhil et al. [7]. However, convenience sampling may introduce selection bias and limit the generalizability of the findings to the wider regional population.

The minimum required sample size was calculated a priori using the Raosoft Sample Size Calculator (Raosoft, Inc., Seattle, WA, USA), yielding an estimated minimum of 384 participants. All eligible records meeting the inclusion criteria during the 24-month study period were subsequently reviewed, and a total of 385 records met this minimum requirement, confirming adequate statistical power based on an anticipated 50% prevalence of VI and blindness (as a conservative estimate in the absence of prior local data), a 95% confidence level, and a 5% margin of error. This calculation used the formula: $n = [z&sup2; &times; p(1-p)] / e&sup2;$

Where n=required sample size, z=1.96 (z-score for 95% confidence), p=0.50 (estimated prevalence), and e=0.05 (margin of error). This calculation yielded an estimated minimum of 384 participants.

Inclusion and exclusion criteria

All patients with complete medical records containing demographic data, medical history, and ophthalmologic examination findings were included. Records with incomplete, unavailable, or unreliable data, including missing key clinical variables such as best-corrected or uncorrected VA (UCVA), laterality, or primary ophthalmologic diagnosis, were excluded. When inconsistencies in VA documentation were identified, the most complete documented ophthalmologic assessment was used, and VI severity was classified according to the VA measurement recorded in the final clinical assessment. Each eligible record was reviewed to document clinical characteristics, causes of VI, and the frequency of VI and blindness.

For binary logistic regression analysis, the dependent variable was coded as 0=no VI or blindness and 1=presence of VI or blindness. Age was entered as a continuous variable, and reference categories for categorical predictors were specified in the regression table.

VA assessment and classification

In clinical records, distance VA was assessed using a Snellen chart at a standard distance of 6 meters under standardized illumination. Both UCVA and best-corrected visual acuity (BCVA) were documented where available. VI was classified according to the WHO International Classification of Diseases, 11th Revision (ICD-11), based on the VA of the better-seeing eye in patients with bilateral involvement and the affected eye in patients with unilateral VI. The severity categories were as follows: no or mild VI (Grade 0), VA ≥ 6/18; moderate VI (Grade 1), VA < 6/18 to ≥ 6/60; severe VI (Grade 2), VA < 6/60 to ≥ 3/60; and blindness (Grades 3-5), VA < 3/60 to no light perception [8].

Definition of refractive error

Refractive error was defined as a spherical equivalent of ≤ -0.50 diopters (D) for myopia, ≥ +0.50 D for hyperopia, and a cylindrical error of ≥0.50 D for astigmatism [9,10].

Data collection and cleaning

Data were extracted from electronic medical records at QUMC by the research team. Each record was reviewed systematically for the following variables: demographic data (age, gender, nationality), presenting complaint, presenting VA (UCVA or BCVA), laterality of impairment, primary diagnosis (cause of VI or blindness), systemic comorbidities, and type of intervention received. Data were entered into a structured Excel spreadsheet (Microsoft Corporation, Redmond, WA, USA) and subsequently imported into SPSS for analysis. Data cleaning involved checking for duplicates, resolving inconsistencies in VA documentation, and verifying that all included records met the eligibility criteria. Records with missing core variables were excluded at this stage. A comprehensive data abstraction sheet was developed to record all study variables. The complete layout of which is available in Appendix 1.

Statistical analysis

Data analysis was performed using IBM SPSS Statistics for Windows, Version 27 (Released 2019; IBM Corp., Armonk, New York, United States). Descriptive statistics, including frequencies and percentages, were used to summarize demographic characteristics and clinical variables. Associations between categorical variables were assessed using the Pearson chi-square test (χ²). For binary logistic regression analysis, the dependent variable was coded as 0=no VI or blindness and 1=presence of VI or blindness. Age was entered as a continuous variable. Reference categories for categorical predictors were as follows: gender (reference=female), nationality (reference=non-Saudi), presenting VA (reference=best-corrected), systemic comorbidities (reference=no comorbidities), and intervention (reference=no intervention received). with results expressed as adjusted odds ratios (AOR) with 95% confidence intervals (CI). A p-value of <0.05 was considered statistically significant. All figures were created using Microsoft PowerPoint (Microsoft Corporation, Redmond, WA, USA).

Ethical considerations

Ethical approval was obtained from the Committee of Research Ethics at Qassim University (Approval No. 25-01-06; dated August 27, 2025), in accordance with the principles of the Declaration of Helsinki [11]. All patient information was kept strictly confidential. No personal identifiers were used in the analysis or reporting, and access to the data was restricted to the research team.

## Results

The study investigated 385 complete medical records of patients of various age groups who underwent ophthalmologic evaluation at QUMC from January 2023 to December 2024. The majority, 337 (87.5%) of the patients were Saudi nationals, with a mean age of 43.56 years and a predominance of males (197, 51.2%) (Table 1).

**Table 1:** Demographic data of the patients (N=385) Data presented as frequencies (n) and proportions (%). n: number of patients.

Demographic data	Category	N (%)
Age	Under 18 years	93 (24.2%)
	18-25	34 (8.8%)
	26-35	34 (8.8%)
	36-45	19 (4.9%)
	46-55	32 (8.3%)
	56-65	77 (20.0%)
	66 years above	96 (24.9%)
Gender	Male	197 (51.2%)
	Female	188 (48.8%)
Nationality	Saudi	337 (87.5%)
	Non-Saudi	48 (12.5%)

**Figure 1:**
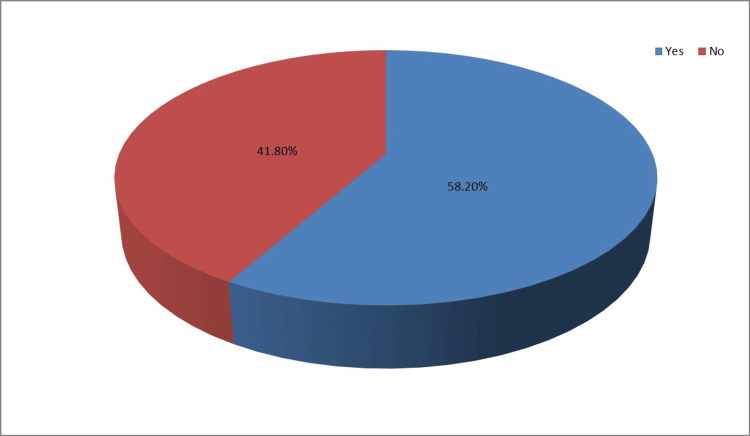
Prevalence of visual impairment and blindness Pie chart illustrating the prevalence of visual impairment and blindness.

Figure 1 illustrates the prevalence of VI and blindness among the patients. The majority of patients, 224 (58.2%), had VI or blindness, while 161 (41.8%) had no VI. 

Table 2 presents the ophthalmologic characteristics of the patients. The most common presenting complaints were decreased vision (125, 32.5%) and blurred vision (103, 26.8%). Patients could have more than one documented presenting complaint; therefore, the complaint categories were not mutually exclusive. Most patients had UCVA (301, 78.2%) and bilateral VI (283, 73.5%). The leading causes of VI or blindness were refractive error (125, 32.5%) and cataract (80, 20.8%).

**Table 2:** Ophthalmologic characteristics of the patients (N=385) Data presented as frequencies (n) and proportions (%). VI: visual impairment.

Characteristics	Category	N (%)
Presenting complaint	Decreased vision	125 (32.5%)
	Blurred vision	103 (26.8%)
	Headache	11 (2.9%)
	Eye pain	6 (1.6%)
	Eye trauma	1 (0.3%)
	Checkup	96 (24.9%)
	Other	56 (14.6%)
	Referred	10 (2.6%)
Presenting visual acuity	Uncorrected	301 (78.2%)
	Best-corrected	84 (21.8%)
Laterality	Bilateral visual impairment	283 (73.5%)
	Unilateral visual impairment	102 (26.5%)
Diagnosis and documented cause of visual impairment or blindness	Refractive error	125 (32.5%)
	Cataract	80 (20.8%)
	Diabetic retinopathy	67 (17.4%)
	Misalignment problem	45 (11.7%)
	Glaucoma	41 (10.7%)
	Age-related macular degeneration	10 (2.6%)
	Other	68 (17.7%)

**Figure 2:**
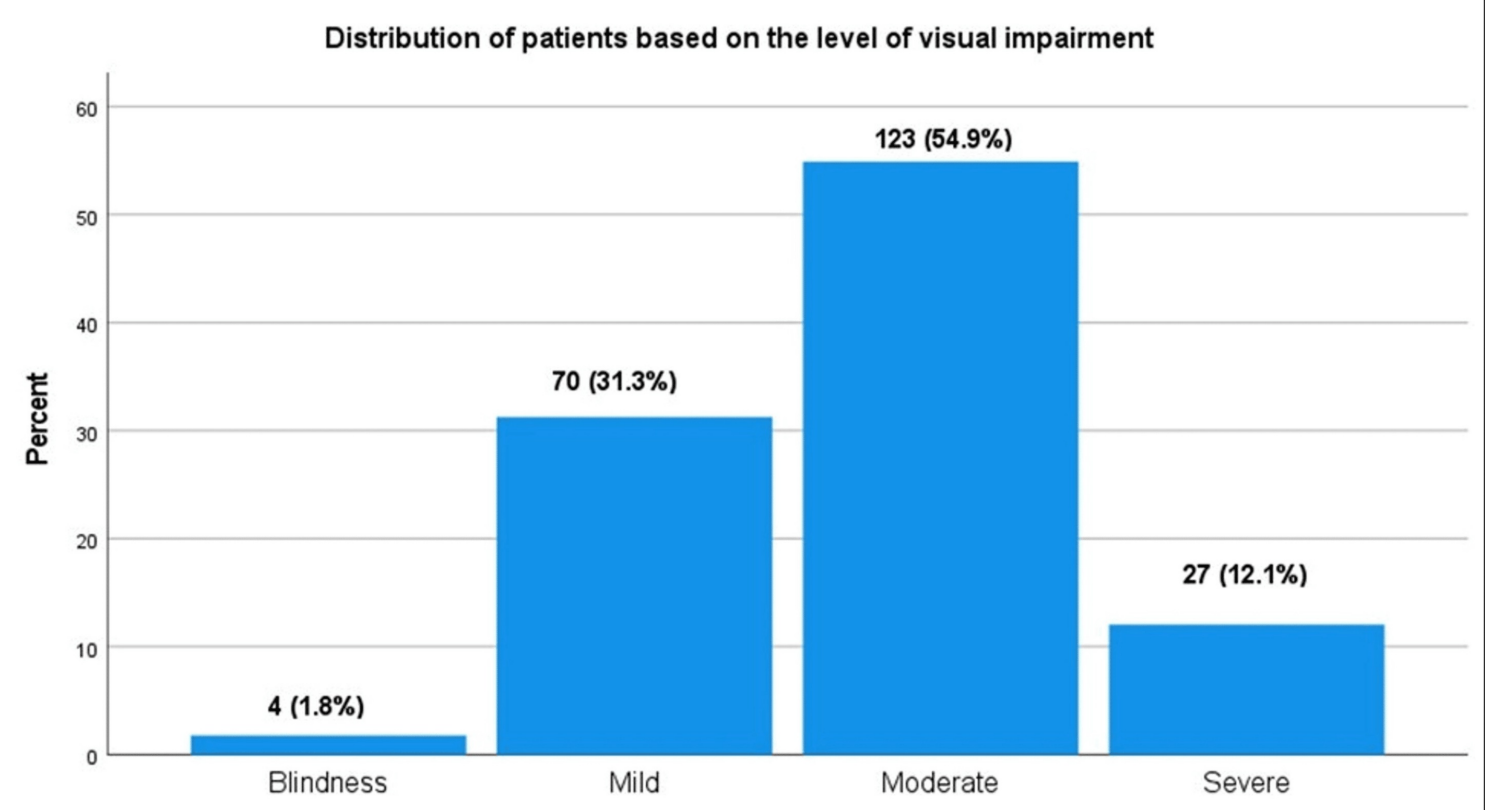
Distribution of patients based on the level of visual impairment Bar chart showing the distribution of patients based on the level of visual impairment and blindness.

Figure 2 presents the distribution of the patients based on the level of VI and blindness. Among the 224 patients with VI or blindness, most had moderate VI (123, 54.9%), followed by mild VI (70, 31.3%), severe VI (27, 12.1%), and blindness (4, 1.8%).

Systemic comorbidities were identified from documented diagnoses in the patients’ electronic medical records (Table 3). The most common comorbidities were diabetes mellitus (159, 74.7%), followed by hypertension (103, 48.4%), cardiovascular disease (38, 17.8%), and neurological disorders (24, 11.3%).

**Table 3:** Systemic comorbidities of the patients (N=385) Data presented as frequencies (n) and proportions (%). Percentages for comorbidity types are calculated among patients with comorbidities (n=213).

Questions	Category	N (%)
Associated with systemic comorbidities	Yes	213 (55.3%)
	No	172 (44.7%)
Specify	Diabetes mellitus	159 (74.7%)
	Hypertension	103 (48.4%)
	Cardiovascular disease	38 (17.8%)
	Neurological disorders	24 (11.3%)
	Others	52 (24.4%)

Table 4 reveals that nearly all patients (94.0%; n=362) used an intervention, primarily through corrective lenses or low-vision aids (219, 60.5%). Most patients (362, 94.0%) had received at least one documented ophthalmic intervention, most commonly corrective lenses or low-vision aids (219, 60.5%).

**Table 4:** Interventions received by the patients (N=385) Data presented as frequencies (n) and proportions (%). Percentages for intervention types are calculated among patients who received an intervention (n=362).

Questions	Category	N (%)
Use of intervention	Yes	362 (94.0%)
	No	23 (6.0%)
Specify	Use of corrective lenses or low-vision aids	219 (60.5%)
	Medical or laser treatment for ocular disease	179 (49.4%)
	History of ocular surgery (e.g. cataract extraction)	130 (35.9%)

Table 5 shows that age, nationality, presenting VA, systemic comorbidities, and intervention status were significantly associated with the presence of VI or blindness (p <0.001*, p=0.030, p<0.001, p<0.001, p=0.005). A higher proportion of older patients, Saudi nationals, patients with uncorrected presenting VA, patients with systemic comorbidities, and patients who had received an intervention had VI or blindness. No significant associations were found between gender, laterality, and the prevalence of VI or blindness (p>0.05).

**Table 5:** Association between demographic and clinical variables and prevalence of visual impairment and blindness (N=385) Data presented as frequencies (n) and proportions (%). Statistical test: Pearson chi-square (χ²). VI: visual impairment. *Statistically significant at p<0.05.

Variables	Prevalence of visual impairment and blindness			
	Category	No	Yes	p-value
Age	Under 18 years	69 (74.2%)	24 (25.8%)	<0.001*
	18-25	19 (55.9%)	15 (44.1%)	
	26-35	26 (76.5%)	8 (23.5%)	
	36-45	11 (57.9%)	8 (42.1%)	
	46-55	13 (40.6%)	19 (59.4%)	
	56-65	14 (18.2%)	63 (81.8%)	
	66 years above	9 (9.4%)	87 (90.6%)	
Gender	Male	85 (43.1%)	112 (56.9%)	0.588
	Female	76 (40.4%)	112 (59.6%)	
Nationality	Saudi	134 (39.8%)	203 (60.2%)	0.030*
	Non-Saudi	27 (56.3%)	21 (43.8%)	
Presenting visual acuity	Uncorrected	104 (34.6%)	197 (65.4%)	<0.001*
	Best-corrected	57 (67.9%)	27 (32.1%)	
Laterality	Bilateral visual impairment	123 (43.5%)	160 (56.5%)	0.276
	Unilateral visual impairment	38 (37.3%)	64 (62.7%)	
Systemic comorbidities	Yes	54 (25.4%)	159 (74.6%)	<0.001*
	No	107 (62.2%)	65 (37.8%)	
Intervention	Yes	145 (40.1%)	217 (59.9%)	0.005*
	No	16 (69.6%)	7 (30.4%)	

The results revealed statistically significant associations between systemic comorbidities and the levels of VI and blindness (p=0.041) (Table 6). A higher proportion of Saudi patients with systemic comorbidities exhibited moderate to severe VI and blindness.

**Table 6:** Association between systemic comorbidities and levels of visual impairment (N=224) Data presented as frequencies (n) and proportions (%). Statistical test: Pearson chi-square (χ²), p=0.041*. *Statistically significant at p<0.05.

Variables	Levels of visual impairment and blindness					
	Category	Mild	Moderate	Severe	Blindness	p-value
Systemic comorbidities	Yes	41 (58.6%)	96 (78.0%)	19 (70.4%)	3 (75.0%)	0.041*
	No	29 (41.4%)	27 (22.0%)	8 (29.6%)	1 (25.0%)	

In the multivariate logistic regression model, intervention received by patients was positively associated with VI and blindness (AOR=3.40; 95% CI=1.219-9.498; p=0.019) (Table 7). Additionally, statistically significant associations were found between age, nationality, presenting VA, systemic comorbidities, intervention status, and the presence of VI or blindness (p<0.001, p=0.030, p<0.001, p<0.001, and p=0.005, respectively.

**Table 7:** Multivariate logistic regression analysis of factors associated with visual impairment and blindness (N=385) AOR: adjusted odds ratio; CI: confidence interval. **Statistically significant at p<0.05.

Variables	AOR	95% CI	P-value
Age	1.05	1.035– 1.065	<0.001**
Gender	1.00	0.610– 1.646	0.994
Nationality	0.55	0.267– 1.152	0.114
Presenting visual acuity	0.42	0.231– 0.756	0.004**
Systemic comorbidities	0.93	0.486– 1.795	0.839
Intervention	3.40	1.219– 9.498	0.019**

## Discussion

Visual health represents a major public health concern, as unaddressed VI can lead to blindness and severely compromise quality of life. Regular eye examination plays a crucial role in early diagnosis and treatment, particularly in Saudi Arabia, where blinding eye diseases remain among the leading causes of disability [12]. The current study aimed to assess the prevalence and causes of VI and blindness in the Qassim region, highlighting patterns that can guide preventive and clinical strategies.

The study revealed a considerably high prevalence of VI, with more than half of the patients (58.2%) exhibiting some degree of vision loss or blindness. A previous study by Alrasheed et al. also documented a substantial burden of VI in the Qassim region [13]. However, direct comparison of prevalence estimates is limited because Alrasheed et al. focused exclusively on children, whereas the present study included patients across a broad age range. Differences in study populations, age distributions, and clinical settings should therefore be considered when interpreting the findings. These findings suggest that the burden of visual health conditions extends across age groups and remains a growing concern. Such high prevalence can be attributed to multiple factors, including limited access to preventive eye care, delayed treatment-seeking behavior, and the increasing incidence of systemic diseases such as diabetes within the Saudi population [6]. The high prevalence observed in this clinical sample also likely reflects a referral bias inherent to tertiary eye-care settings, where patients presenting to QUMC may have more advanced disease compared to the general population.

Most patients presented with moderate VI (54.9%), followed by mild (31.3%) and severe forms (12.1%), with blindness accounting for 1.8% of cases. These findings correspond with reports from Ethiopia [14] and India [15], where the majority of affected individuals also had moderate impairment. This finding is consistent with the severity distribution reported in these studies, where moderate VI was also the most common category, although methodological and population differences should be taken into account. The predominance of moderate cases in this study underscores the opportunity for early intervention and rehabilitation to prevent progression to irreversible blindness.

Decreased vision (32.5%) and blurred vision (26.8%) were the most frequent complaints, while refractive error (32.5%) and cataract (20.8%) emerged as the leading causes of vision loss. These findings align with the observations by Khathami et al. [16], who identified uncorrected refractive error and cataract as principal causes of VI and blindness in Saudi Arabia. Given that refractive error and cataract are treatable conditions, their frequent occurrence in this clinic-based sample emphasizes the need for early identification, timely refractive correction, and appropriate cataract management, especially among older adults and patients at higher risk of vision loss. Regular community-based screening and subsidized optical services could substantially reduce these preventable cases.

Most patients (55.3%) had systemic comorbidities, including diabetes mellitus (74.7%) and hypertension (48.4%), consistent with the studies by Choi et al. [17] and Kahloun et al. [18], which reported hypertension and diabetes as common systemic comorbidities associated with VI and blindness. Furthermore, a higher proportion of patients with systemic comorbidities exhibited moderate to severe VI and blindness (p=0.041). This association may be explained by the ocular complications of chronic systemic diseases. Diabetes can contribute to VI through diabetic retinopathy, diabetic macular edema, and retinal microvascular damage [19,20], while hypertension may contribute through hypertensive retinopathy and retinal vascular changes [21]. However, because of the retrospective cross-sectional design of this study, causal relationships cannot be established. These findings underscore the importance of integrated management of systemic diseases as a key preventive strategy for VI.

An overwhelming majority of patients (94.0%) had received an intervention, most commonly corrective lenses or low-vision aids (60.5%). Additionally, intervention was positively associated with VI and blindness (AOR=3.40; 95% CI=1.219-9.498; p=0.019). This paradoxical finding is likely explained by reverse causality: patients with established VI are more likely to seek and receive interventions. Rather than implying that interventions worsen vision, this association confirms that intervention use is a marker of disease severity in the study population. Age was also independently associated with VI (AOR=1.05 per year; p<0.001), consistent with the known age-related progression of cataracts, macular degeneration, and glaucoma [4,14].

Limitations

This study has several limitations. First, because of its retrospective design, the analysis depended on the completeness and accuracy of medical-record documentation. Second, the study was conducted at a single tertiary-care center using a clinic-based sample, which may introduce selection and referral bias, as patients attending ophthalmology clinics may have more advanced or symptomatic disease than the general population. Therefore, the findings should not be interpreted as population-based estimates for the Qassim region. Third, convenience sampling may limit the generalizability of the results. Fourth, in patients with multiple ocular diagnoses, assigning a single primary cause of VI may have introduced misclassification. Finally, the positive association between intervention status and VI should be interpreted cautiously, as it may reflect reverse causality, whereby patients with more severe VI were more likely to receive ophthalmic interventions.

This study provides clinically relevant data on VI and blindness among patients attending a tertiary ophthalmology clinic in the Qassim region. However, its retrospective design and dependence on medical-record documentation should be considered when interpreting the findings.

Future directions

Future studies should consider population-based cohort designs with random sampling across multiple sites within the Qassim region to improve generalizability. Longitudinal follow-up would allow assessment of disease progression and treatment outcomes. Investigations incorporating genetic, nutritional, and environmental risk factors would provide a more comprehensive understanding of the determinants of visual impairment in the Saudi context. Community-based vision screening programs and their impact on early detection and referral patterns also merit prospective evaluation.

## Conclusions

VI and blindness were common among patients attending ophthalmology clinics at QUMC. These findings should be interpreted within the context of a single-center, clinic-based sample and should not be generalized to the broader Qassim population. The most frequent presenting symptoms were decreased vision and blurred vision. Refractive error and cataract were the leading documented causes, and most affected patients had moderate or mild VI. Diabetes mellitus and hypertension were the most common systemic comorbidities. These findings highlight the importance of regular eye examinations, timely refractive correction, appropriate cataract management, and integrated care for systemic diseases among clinic attendees. Further population-based studies are needed to determine the broader burden of VI and blindness in the Qassim region.

## Appendices

Appendix 1

Questionnaire

Section 1: Demographic data

1. Age

2. Gender:

Male

Female

3. What is the nationality?

Saudi

Non-Saudi

Section 2: Ophthalmologic characteristics  

1. Presenting complaint

Blurred vision

Low vision

Eye trauma

Headache

Eye pain

Referred

Checkup

Other

2. Visual acuity 

3. Presenting visual acuity 

Uncorrected

Best-corrected

4. Laterality 

Unilateral visual impairment

Bilateral visual impairment

5. Diagnosis and documented cause of visual impairment or blindness  

 Cataract

Diabetic retinopathy

Glaucoma

Refractive error

Age-related macular degeneration

Misalignment problem

Other

Section 3: Systemic comorbidities (if available)

1. Are there systemic comorbidities?

Yes

No

2. If "Yes" Specify

Diabetes mellitus

Hypertension

Cardiovascular disease

Neurological disorders

Other

Section 4: Interventions (if recorded)

1. Is there an intervention?

Yes

No

2. If "yes," specify

History of ocular surgery (e.g., cataract extraction)

Medical or laser treatment for ocular disease

Use of corrective lenses or low vision aids

Other
